# The role of proteinaceous toxins secreted by *Staphylococcus aureus* in interbacterial competition

**DOI:** 10.1093/femsmc/xtae006

**Published:** 2024-02-28

**Authors:** Stephen R Garrett, Tracy Palmer

**Affiliations:** Newcastle University Biosciences Institute, Newcastle University, Newcastle upon Tyne NE2 4HH, United Kingdom; Newcastle University Biosciences Institute, Newcastle University, Newcastle upon Tyne NE2 4HH, United Kingdom

**Keywords:** *Staphylococcus aureus*, bacteriocin, T7SS, interbacterial, competition, toxin, host colonization

## Abstract

*Staphylococcus aureus* is highly adapted to colonization of the mammalian host. In humans the primary site of colonization is the epithelium of the nasal cavity. A major barrier to colonization is the resident microbiota, which have mechanisms to exclude *S. aureus*. As such, *S. aureus* has evolved mechanisms to compete with other bacteria, one of which is through secretion of proteinaceous toxins. *S. aureus* strains collectively produce a number of well-characterized Class I, II, and IV bacteriocins as well as several bacteriocin-like substances, about which less is known. These bacteriocins have potent antibacterial activity against several Gram-positive organisms, with some also active against Gram-negative species. *S. aureus* bacteriocins characterized to date are sporadically produced, and often encoded on plasmids. More recently the type VII secretion system (T7SS) of *S. aureus* has also been shown to play a role in interbacterial competition. The T7SS is encoded by all *S. aureus* isolates and so may represent a more widespread mechanism of competition used by this species. T7SS antagonism is mediated by the secretion of large protein toxins, three of which have been characterized to date: a nuclease toxin, EsaD; a membrane depolarizing toxin, TspA; and a phospholipase toxin, TslA. Further study is required to decipher the role that these different types of secreted toxins play in interbacterial competition and colonization of the host.

## Teaser

A review on antibacterial proteins and peptides secreted by *Staphylococcus aureus* that are used to kill competing bacteria.

*Staphylococcus aureus* is an opportunistic human pathogen capable of causing disease at many sites in the body. This is mediated by an abundance of virulence factors, which allow the bacterium to invade host tissues and effectively evade the immune system (reviewed in Thammavongsa et al. 2015, Howden et al. 2023). *S. aureus* infections most commonly occur in immunocompromised individuals, with the bacterium usually entering the bloodstream through a breach in the skin barrier, and can be life-threating. While *S. aureus* infections are prevalent, particularly within healthcare settings, *S. aureus* disease is an outcome for only a very small proportion of individuals colonized by these bacteria, with around 30% of the population colonized at any one time (Wertheim et al. 2005). *S. aureus* has evolved a number of mechanisms to enable successful colonization of the host including immune evasion and nutrient acquisition strategies, and biosynthesis of adhesins that recognize human cell surface receptors. However, one of the first barriers *S. aureus* must overcome during colonization is the resident microbiota.

Whilst *S. aureus* can invade and persist in almost every niche within the human host, it is most commonly found to colonize the nasal cavity, vaginal tract and skin (Dancer 2008). The anterior nares are a highly competitive environment, which can harbour diverse bacterial species (Liu et al. 2015, Schenck et al. 2016, Kumpitsch et al. 2019). Commensal bacteria are highly adapted to compete with *S. aureus*, controlling *S. aureus* populations through production of antagonistic autoinducing peptides (Severn et al. 2022, Williams et al. 2023), release of antibacterial compounds such as antibiotics and bacteriocins (Newstead et al. 2020, Zhao et al. 2022), and by competing for adhesion sites (Maciag et al. 2023). *S. aureus* has, therefore, evolved counter measures to outcompete the resident microbiota to enable colonization of the host. *S. aureus* can compete with commensals for binding sites within the host, expressing a large number of surface-exposed adhesin-like molecules, which have incredibly high affinity for host factors (Foster et al. 2014, Sakr et al. 2018). *S. aureus* is also highly adapted to the nutrient-poor environment of the nasal cavity through upregulation of several nutrient uptake systems (Krismer et al. 2017). However, *S. aureus* can also shape the microbiota by the secretion of antibacterial substances.

Antibiotics and bacterial toxins that are secreted by commensal organisms for the control of *S. aureus* infections have been studied extensively (Newstead et al. 2020, Heinzinger et al. 2023). By contrast, less is understood about antibacterial compounds secreted by *S. aureus* and the role that these play in colonization of the host, which will be the focus of this review.

## *S. aureus* secreted bacteriocins

Bacteriocins are antimicrobial proteins, or peptides, that are released by bacteria usually to target closely related bacterial species (Riley and Wertz 2002, Heilbronner et al. 2021). Bacteriocins can range from large proteins such as colicins and pyocins, to small peptides less than 5 kDa in size. Large bacteriocins such as colicins and pyocins are commonly produced by Gram-negative bacteria and usually require cell lysis for their release (Michel-Briand and Baysse 2002, Cascales et al. 2007). By contrast, bacteriocins produced by Gram-positive bacteria are commonly secreted from the cell by the general secretory pathway or by specialized transport machineries (Ennahar et al. 2000, Gajic et al. 2003, Ishibashi et al. 2014). In addition, Gram-positive bacteriocins often have dedicated regulatory pathways, decoupling host fate from bacteriocin production (Ennahar et al. 2000, Riley and Wertz 2002). A likely result of this is the large diversity of bacteriocins produced by Gram-positive bacteria (Jack et al. 1995, Acedo et al. 2018).

Bacteriocins produced by staphylococcal species can be categorized into one of six groups. The majority of staphylococcal bacteriocins are Class I bacteriocins, which are small (< 5 kDa) heat-stable peptides that are post-translationally modified, and include groups such as lantibiotics (Bierbaum and Sahl 2009). Class II bacteriocins are also small (< 10 kDa) heat-stable peptides, which are not post-translationally modified (Nissen-Meyer et al. 2009). Class III bacteriocins are much larger (> 30 kDa) proteins, which are heat-labile and can be subdivided as either lytic or non-lytic (Heng et al. 2007). Class IV molecules are post-translationally modified cyclic peptides (Van Belkum et al. 2011).

More recent discoveries have identified two further bacteriocin groups, consisting of sanctipeptides and thiopeptides (Varella Coelho et al. 2017, Zheng et al. 2017). Whilst these have not yet been formally classified, it has been proposed that they constitute Class V and Class VI, respectively (De Freire Bastos et al. 2020). A recent review suggested that the classification system for bacteriocins be simplified to two classes—either modified or unmodified peptides (Soltani et al. 2021). However, for consistency we will continue to use the classification used in (Newstead et al. 2020), which also covered staphylococcal bacteriocins.

It is thought that up to 99% of all bacteria produce at least one bacteriocin (Klaenhammer 1988), meaning there is a plethora of as yet unidentified antimicrobial compounds. Numerous studies have identified bacteriocin-like inhibitory substances (BLIS), which are secreted by e.g. staphylococcal species, but unlike bacteriocins, the genetic origin, chemical structure, and mode of action remain poorly characterized (James and Tagg 1991). Whilst bacteriocins and BLIS can be found as secreted products across staphylococcal species, to date, only Class I, II, and IV bacteriocins have been shown to be secreted by *S. aureus*. Upwards of 15 different bacteriocins and BLIS have now been identified as being produced by *S. aureus*, with seven of these currently classified.

## *S. aureus* Class I bacteriocins

**Figure 1.**
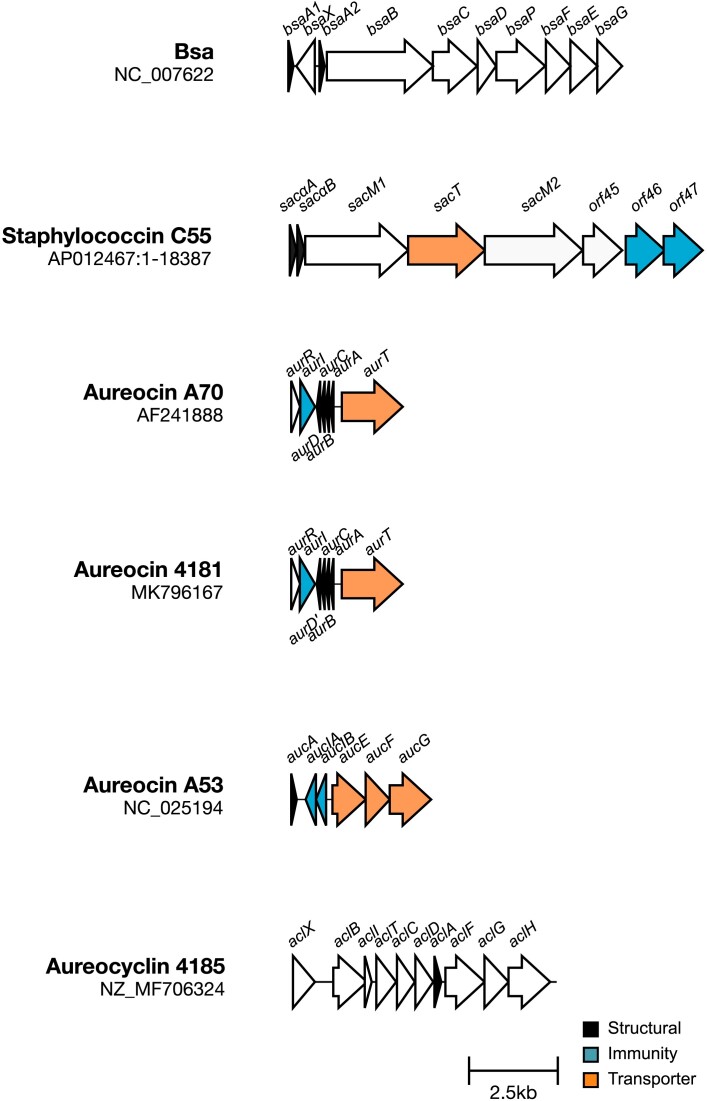
Biosynthetic clusters of *S. aureus*-produced bacteriocins. Known biosynthetic clusters of *S. aureus* bacteriocins. Bacteriocin structural genes, immunity-encoding genes, and known transporter-encoding genes are highlighted. Gene diagrams were visualized using Clinker (Gilchrist and Chooi 2021).

**Figure 2.**
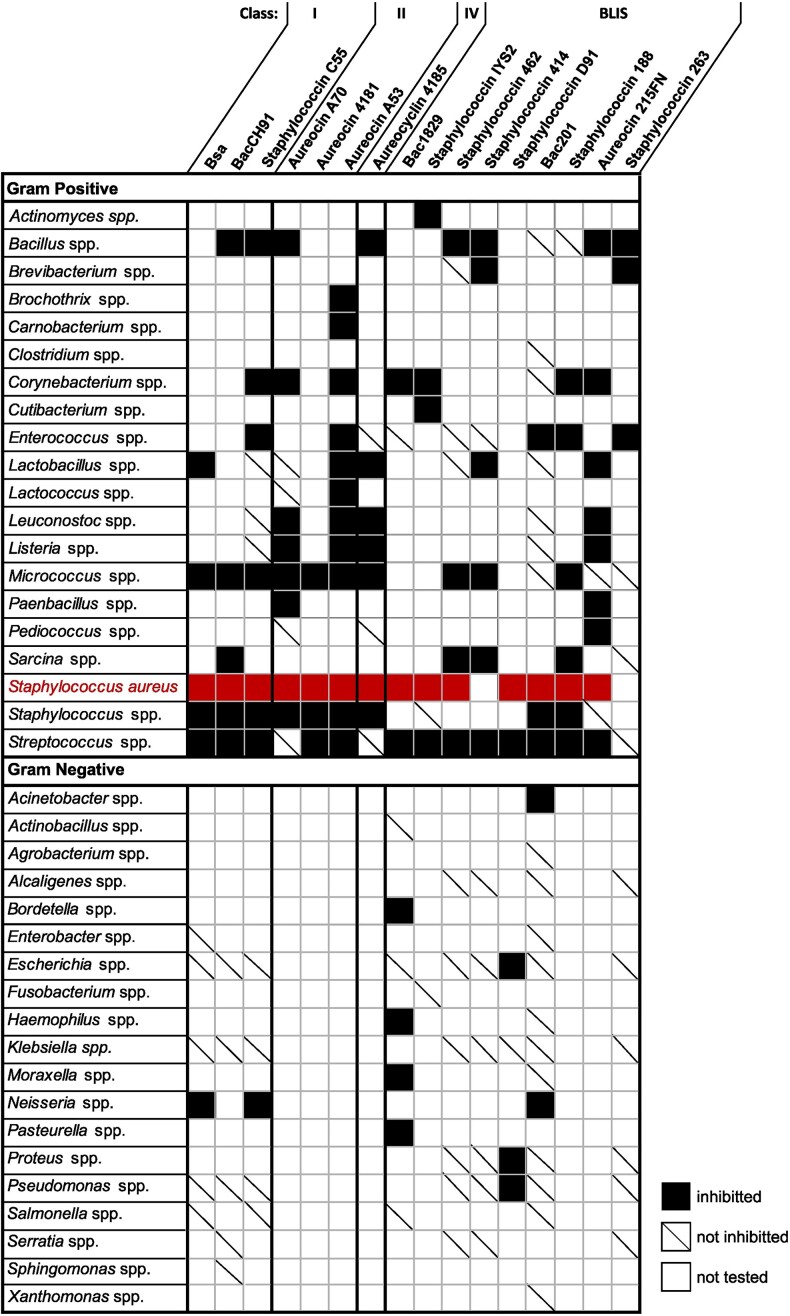
Bacteriocins secreted by *S. aureus* strains. A summary of all *S. aureus*-secreted bacteriocins and the genus of bacteria that they have been shown to inhibit. Shading represents sensitivity of species within a given genera to the *S. aureus* produced bacteriocin and a diagonal dash represents resistance, within a genera, to the bacteriocin. A blank space indicates that a given bacteriocin has not been tested against any species of the corresponding genera. As the producer of these bacteriocins, *S. aureus* (highlighted in red), has been included separately from other staphylococcal species, to highlight the activity of *S. aureus*-produced bacteriocins against other *S. aureus* strains.

In 1992, a BLIS was identified in the supernatant of *S. aureus* strain 26, which could inhibit the growth of *Streptococcus pyogenes* and *Micrococcus luteus*, and so was named staphylococcin Au-26 (Scott et al. 1992). This was later identified as the Class I bacteriocin, Bsa. Bsa is a 21-amino acid lantibiotic encoded downstream of *lukD* on the Type II νSaβ genomic island (Daly et al. 2010) (Fig. 1). Up to two nonidentical copies can be encoded at this locus, *bsaA1* and *bsaA2*, followed by the required biosynthetic gene cluster. The *bsa* gene cluster and the Bsa peptide sequence are similar to the epidermin family of lantibiotics (Daly et al. 2010). Of these gene products, BsaBCD are predicted to be involved in post-translational modification of Bsa, and BsaEFG are likely involved in providing immunity to this bacteriocin, although this has yet to be confirmed (Daly et al. 2010). It was further shown that Bsa has activity against *M. luteus* amongst other bacterial species (Scott et al. 1992, Daly et al. 2010) (Fig. 2). However, the antibacterial activity observed by Daly et al. (2010) was based on a peptide that was 2 Da smaller than that predicted for BsaA2. It has since been shown that the antimicrobial activity observed in this assay was due to phenol soluble modulins (PSMs) (Joo et al. 2011), which are secreted by *S. aureus* and have previously been found to have cytolytic effects on eukaryotic cells (Cheung et al. 2014). Nevertheless, Bsa was found to have antimicrobial activity and appears to be a Class I lantibiotic (Scott et al. 1992).

A further Class I bacteriocin produced by *S. aureus* has been characterized. BacCH91 was isolated from the supernatant of *S. aureus* CH91, following observed bacteriocin-like activity. N-terminal Edman degradation and chemical derivatization of the bacteriocin allowed for the identification of the 21-residue amino acid sequence, identical to that of BsaA2 from strain ET3-1 (Daly et al. 2010, Wladyka et al. 2013). Unlike the Bsa study described above, BacCH91 was purified prior to sequencing and subsequent assays, and therefore the activity observed is unlikely to be due to alternative antimicrobial molecules. Whilst no activity was observed against Gram-negative bacteria, BacCH91 had potent antibacterial activity against most Gram-positive bacteria tested, including *M. luteus, Streptococcus* spp., and several staphylococcal species (Fig. 2). Interestingly, BacCH91 was also able to inhibit the growth of *S. aureus* CH91, the producing strain. This is uncommon for bacteriocins as the producing strain is often either naturally immune or has a cognate immunity mechanism for protection (Cotter et al. 2005, Pérez-Ramos et al. 2021).

Whilst staphylococcin Au-26, Bsa, and BacCH91 are all likely to be closely related antimicrobial peptides, it is now clear that these Class I lantibiotics have potent activity against several Gram-positive bacteria, in particular those that are found to commonly colonize the human skin and nasal passages.

Staphylococcin C55 is another Class I bacteriocin produced by *S. aureus* strains. Staphylococcin C55 has antibacterial activity against many strains of *S. aureus* and against some coagulase-negative staphylococci (Navaratna et al. 1998). Whilst C55α has low levels of activity alone, activity increases 128-fold in the presence of an equimolar ratio of C55β (Navaratna et al. 1998). C55α and C55β contain lanthionine, which is post-translationally modified, likely mediated by C55M1, a protein encoded by *sacM1* found downstream of the C55 peptide-encoding genes (*sacαA* and *sacβA*), and which shares homology to other lantibiotic modification proteins (Navaratna et al. 1999) (Fig. 1). A putative lantibiotic transporter (SacT) is also encoded in this operon. The staphylococcin C55 operon is encoded on a 32-kB plasmid, which has been shown to protect the producer strain from killing by this bacteriocin (Navaratna et al. 1998). Two open reading frames encoded downstream of this operon are important for immunity against staphylococcin C55, which explains why *S. aureus* strains that do not carry this plasmid are susceptible to killing (Kawada-Matsuo et al. 2016).

A BLIS, BacR1, identified from *S. aureus* U0007 has previously been shown to have activity against a wide range of Gram-positive bacteria, and against the Gram-negative *Neisseria gonorrhoeae* (Rogolsky and Wiley 1977, Morriss et al. 1978) (Fig. 2). It has since been found that BacR1 is identical to the C55α of staphylococcin C55, both produced from the same plasmid in different strains (Navaratna et al. 1998, 1999). It is possible that this could also be the case for other BLIS currently identified.

## *S. aureus* Class II bacteriocins

Class II bacteriocins represent unmodified bacteriocins, of which three have been identified to date in *S. aureus*: aureocin A53, aureocin A70, and aureocin 4181.

A study of *S. aureus* strains isolated from commercial milk identified several BLIS, which were also found to be encoded on plasmids. Based on the data available, at least two different plasmids encoding phenotypically diverse BLIS have been identified (Giambiagi-Marval et al. 1990). It was found that BLIS produced from plasmids in *S. aureus* A53 and A70 strains were both able to inhibit *Listeria* spp., *Corynebacterium fimi, Micrococcus sp*., and other *S. aureus* strains, which did not harbour the plasmids (De Oliveira et al. 1998a) (Fig. 2). In addition *S. aureus* A53 was also able to inhibit the growth of *Lactobacillus* spp., *Lactococcus lactis*, and *Streptococcus* spp. (De Oliveira et al. 1998a). The bacteriocins responsible for the activity observed have since been characterized as members of the Class II family.

Aureocin A70 is produced by *S. aureus* A70, carrying the pRJ6 plasmid, which harbours the aureocin A70 peptide-encoding genes and associated genes required for its production and secretion (Giambiagi-Marval et al. 1990, Netz et al. 2001). Aureocin A70 is composed of four small, related peptides, AurABCD, which are strongly cationic and highly hydrophobic (Netz et al. 2001) (Fig. 1). It has been shown that unlike staphylococcin C55, the aureocin A70 peptides are not modified prior to secretion (Netz et al. 2001). Interestingly, at least three of these peptides (AurABC) have antimicrobial activity alone, in the absence of other aureocin A70 peptides. AurD is likely to have similar activity, however, could not be purified (Netz et al. 2001). Aureocin A70 is secreted by an ABC transporter, AurT. Exporters such as this can be used in the efflux of toxic compounds from the cell, however, AurT was found to be dispensable for immunity against this bacteriocin. Instead, AurI, a putative immunity protein, provides immunity against aureocin A70 activity (Coelho et al. 2014). AurI is encoded in a two gene operon, downstream of a transcriptional regulator gene *aurR*. AurR downregulates aureocin A70 production specifically when cells are grown on solid media (Coelho et al. 2016). Aureocin A70 has activity against a wide range of Gram-positive bacteria tested (Giambiagi-Marval et al. 1990, Nascimento et al. 2005, 2006, Varella Coelho et al. 2007, Brito et al. 2011), including against species of *Staphylococcus* and *Corynebacterium*, which commonly colonize the nasal cavity (Fig. 2; Table S1, Supporting Information).

A novel bacteriocin with high similarity to aureocin A70 has since been characterized. Aureocin 4181 is composed of three peptides, which are identical to aureocin A70 AurABC (Fig. 1). AurD, however, carries a Leu to Phe substitution at residue 29 and in addition, all four aureocin 4181 peptides are *N*-formylated (Salustiano Marques-Bastos et al. 2020). Aureocin 4181 was found to have 2–4-fold higher activity than aureocin A70 and could inhibit streptococci, which was not previously observed for aureocin A70 (Marques-Bastos et al. 2020, Salustiano Marques-Bastos et al. 2020, De Oliveira et al. 1998a) (Fig. 2). Nevertheless, neither aureocin could kill strains, which were producing the other bacteriocin, suggesting cross-immunity (Salustiano Marques-Bastos et al. 2020).

Aureocin A53 is produced by *S. aureus* A53 carrying the pRJ9 plasmid (Giambiagi-Marval et al. 1990). A single structural gene, *aucA*, encodes this 6-kDa bacteriocin (Netz et al. 2002) (Fig. 1). Much like aureocin 4181, the bacteriocin has a *N*-formylmethionine at its N-terminus (Netz et al. 2002, Fagundes et al. 2016, Marques-Bastos et al. 2023). Interestingly, genes relating to bacteriocin biosynthesis and modification do not appear to be present on this plasmid. This may be explained by the fact that aureocin A53 is not modified post-translationally, and is highly structured in solution, which has not been observed for amphiphilic bacteriocins previously. As such, aureocin A53 is suggested to share structural similarity with the eukaryotic antimicrobial defensin peptides (Netz et al. 2002). Aureocin A53 is exported by an ABC transporter formed of AucE, AucF, and AucG (Fig. 1). Whilst this can provide some immunity against this bacteriocin, two immunity proteins AucIA and AucIB are required to prevent killing of *S. aureus* by aureocin A53 (Nascimento et al. 2012). Aureocin A53 causes membrane damage, which leads to eventual lysis of the target cell (Netz et al. 2002). The structure of AucA was elucidated by nuclear magnetic resonance, showing that AucA is composed of four short helices, with the outward facing residues being highly cationic, shielding the hydrophobic core (Acedo et al. 2016). It is likely the hydrophobic core is responsible for the membrane damaging activity of aureocin A53. Aureocin A53 has activity against a wide range of Gram-positive organisms, including *Listeria, Enterococcus*, and *Streptococcus* species (Giambiagi-Marval et al. 1990, Netz et al. 2002, Nascimento et al. 2006, Varella Coelho et al. 2007, Acedo et al. 2016) (Fig. 2; Table S1, Supporting Information).

Intriguingly, it was discovered that when aureocin A53 and aureocin A70 were used in combination, activity was much greater then when each were used singly (Varella Coelho et al. 2007). In addition, combination use resulted in toxicity against an *S. aureus* strain, which was resistant to each bacteriocin when used individually. Whilst this may have implications in the development of bacteriocins for therapeutic purposes, further work is required to understand these observations at a molecular level (Varella Coelho et al. 2007). Two recent studies have investigated the development of resistance of *L. lactis* and *Enterococcus faecium* to aureocin A53. It was found that missense mutations in genes coding for the KinG-LlrG two-component system and the LiaFSR stress response components, respectively, conferred resistance to aureocin A53 in these organisms (Tymoszewska et al. 2021, 2023). Further studies will be required to understand the therapeutic potential of bacteriocins and to understand how we can minimize the risk of resistance developing.

## *S. aureus* Class IV bacteriocins

Class IV bacteriocins are post-translationally modified peptides, which have been circularized by covalent linkage of the N- to C-terminus. The supernatant of *S. aureus* 4185 was found to have bacteriocin-like activity, with peptide fragments responsible for such activity identified by mass spectrometry (Ceotto et al. 2010). The BLIS produced by this strain has activity against *M. luteus* and *Listeria monocytogenes*, making these products of potential interest as food preservatives (Ceotto et al. 2010) (Fig. 2; Table S1, Supporting Information). One of the bacteriocins believed to be responsible for this activity is encoded on a plasmid harboured by *S. aureus* 4185 (Potter et al. 2014). Unfortunately this bacteriocin could not be purified, however, *in silico* analysis found the encoding biosynthetic cluster to share high homology with that of carnocyclin A from *Carnobacterium maltaromaticum* (Potter et al. 2014). Carnocyclin A is a cyclic bacteriocin with activity against a diverse range of Gram-positive bacteria (Zipperer et al. 2016). As such, the 60 residue, homologous bacteriocin from *S. aureus* 4185 was named aureocyclin 4185, and is the first putative cyclic bacteriocin identified in *S. aureus* (Potter et al. 2014) (Fig. 1).

Aureocyclin 4185 is predicted to have a short leader peptide, which is cleaved, allowing for the covalent linkage of the N- and C-termini (Potter et al. 2014). Homology modelling suggests that the bacteriocin is composed of four short helices, enclosing a highly hydrophobic core. Several Lys residues likely provide the molecule with the positive charge required for attraction to the bacterial membrane (Potter et al. 2014). Further work is, however, required to confirm that aureocyclin 4185 is responsible for the antibacterial activity imparted by this strain.

## *S. aureus* BLIS

Due to the complex biosynthesis pathways of many bacteriocins, the identification of novel inhibitory substances has often been carried out phenotypically from culture or culture supernatant, rather than through genetic analysis of putative bacteriocin genes. As a result, inhibitory products commonly go uncharacterized due to difficulties in the identification process, and this is the case for many BLIS. Up to eight further *S. aureus* produced bacteriocins have been identified, in addition to those discussed above, however, they remain poorly characterized (Table S1, Supporting Information).

Some BLIS, such as Bac1829, Bac201, and staphylococcin IYS2, have been partially characterized, with the amino acid composition known, but lack the amino acid sequence and subsequent classification. Bac1829 is a 6.4-kDa peptide with a high proportion of hydrophobic residues that has a bactericidal effect on target cells (Crupper and Iandolo 1996, 1997). Staphylococcin IYS2 is a peptide of about 5 kDa, i.e. also bactericidal, and for which the amino acid composition is also known (Nakamura et al. 1983). Both of these BLIS have activity against a wide range of Actinomycetota and other Gram-positive organisms, but Bac1829 also has activity against several Gram-negative bacterial species (Table S1, Supporting Information; Nakamura et al. 1983, Crupper and Iandolo 1996) (Fig. 2). Bac201 is a much larger bacteriocin, comprising a 41-kDa peptide (Iqbal et al. 1999, 2001). The amino acid composition of Bac201 was found to be similar to that of Bac1829, containing of a high proportion of glycine, proline, and alanine residues (Iqbal et al. 2001), however, the amino acid sequence remains unknown. Again, much like Bac1829, Bac201 had activity against a range of both Gram-positive and Gram-negative bacteria tested (Fig. 2; Table S1, Supporting Information), and given the temperature and pH stability of this bacteriocin, may warrant further study for therapeutic development (Iqbal et al. 1999, 2001).

Several BLIS have been purified from the producing *S. aureus* strains, but the amino acid composition remains unknown. Staphylococcin 462 was purified from *S. aureus* 462 and shown to be a peptide of ~9 kDa (Gagliano and Hinsdill 1970, Hale and Hinsdill 1973). Staphylococcin 414 was found to be much larger, migrating in the void volume after size exclusion chromatography, implying a size of over 200 kDa (Gagliano and Hinsdill 1970). This BLIS appeared to be a lipoprotein–carbohydrate complex that was purified from cell lysate rather than culture supernatant. Staphylococcin 414 appears to target Gram-positive organisms exclusively (Gagliano and Hinsdill 1970) (Fig. 2; Table S1, Supporting Information). Staphylococcin D91 is another BLIS that could be purified from its producing strain, which has bacteriostatic activity against a range of Gram-positive and Gram-negative organisms (Kader et al. 1984) (Fig. 2; Table S1, Supporting Information). Staphylococcin D91 is also likely to be plasmid encoded as *S. aureus* D91 loses the ability to produce staphylococcin D91 when grown at 44°C, suggesting the loss of a plasmid encoding this BLIS (Kader et al. 1984, Iqbal et al. 1999). As a sequence and tertiary structure has not been elucidated for any of these BLIS it is not possible for these to be classified. However, given the difference in size and target range of these bacteriocins it suggests that at least some of these are unique, uncharacterized bacteriocins.

The identification of some BLIS, however, has been based solely on activity of *S. aureus* culture supernatant. Antibacterial activity was observed for staphylococcin 188 from the supernatant of *S. aureus* 188, however, this was solely observed against Gram-positive and Actinomycetota species (Saeed et al. 2004) (Fig. 2; Table S1, Supporting Information). Likewise, aureocin 215FN from culture supernatant, had activity against Gram-positive species only (Nascimento et al. 2006, Varella Coelho et al. 2007, De Oliveira et al. 1998b).

A recent bioinformatic study has identified biosynthetic clusters in *S. aureus* strains for both lactococcin 972 and micrococcin P1, suggesting there are additional *S. aureus*-produced bacteriocins yet to be identified. Whilst much work has been carried out to identify the bacteriocins that are secreted by *S. aureus*, the mechanism by which they inhibit growth remains poorly understood for many of these. Nonetheless, it is clear that *S. aureus*-produced bacteriocins can have a wide range of targets and are likely used in colonization, to outcompete resident microbes, and also to exclude unwanted organisms once established. However, the sporadic distribution of these antimicrobial compounds suggest they may not be a widespread mechanism used by *S. aureus* to target competitors.

## A role for PSMs in bacterial killing

PSMs are small peptides secreted by staphylococcal species, which have cytolytic activity against eukaryotic host cells (Peschel and Otto 2013). However, there is some evidence that PSMs can also be used to kill competing bacteria. PSMδ produced by *S. epidermidis* can inhibit the growth of *S. pyogenes* on murine skin (Cogen et al. 2010), although concentrations required to mediate inhibition are very high, suggesting this may not be the evolved purpose of these PSMs. The *S. aureus* strain, USA300 has high levels of antimicrobial activity against *S. pyogenes* and *M. luteus*, which is modulated by PSMα and PSMβ (Joo et al. 2011). However, this activity is only observed when PSMα and PSMβ are proteolytically processed at the N-terminus. It has been suggested that this processing is carried out by microbial exoproteases, indicating that targeting of host and bacterial cells may not always be mutually exclusive. Nevertheless, some PSMs appear to be able to kill the Group A *Streptococcus, S. pyogenes*, possibly playing a role in controlling this pathogen within the host. PSMs do not have activity against staphylococcal species due to the presence of the phenol-soluble modulin ABC transporter, responsible for the export of PSMs from the cell (Chatterjee et al. 2013).

## The Bacillota type VII secretion system and interbacterial competition

Alongside the study of bacteriocins, a growing field of research has started to unravel roles in interbacterial competition of a protein export pathway encoded by all *S. aureus* strains, named the Type VII Secretion System (T7SS). The T7SS was first identified in *Mycobacterium* species, as the system responsible for the secretion of the potent virulence factor, ESAT-6 (Pym et al. 2002, Guinn et al. 2004). Structural analysis of a mycobacterial T7SS named ESX-5 shows that it forms a large membrane complex with a hexameric arrangement of a membrane-bound ATPase, EccC, at the centre, forming a translocation pore (Beckham et al. 2021, Bunduc et al. 2021). A distant homologue of the mycobacterial system is found in many Bacillota (previously Firmicutes), including *S. aureus* and has been designated the T7SSb.

The T7SSb comprises four conserved membrane proteins that likely form a complex. Based on sequence conservation between mycobacterial EccC and the T7SSb EssC component, it is likely that a hexamer of EssC forms the central pore, facilitating substrate export across the membrane, driven by ATP hydrolysis (Burts et al. 2005, Rosenberg et al. 2015, Zoltner et al. 2016, Klein et al. 2021). The T7SSb of some Bacillota is required for full virulence, with *ess/T7SSb* mutant strains of *Streptococcus* and *S. aureus* attenuated in murine and zebrafish models of infection (Burts et al. 2005, Anderson et al. 2011, Kneuper et al. 2014, Dai et al. 2017, Ulhuq et al. 2020, Taylor et al. 2021, Spencer and Doran 2022, Schindler et al. 2023). However, in other species, such as *L. monocytogenes*, the T7SSb was found to play no detectable role in virulence (Way and Wilson 2005, Pinheiro et al. 2017). In recent years, a growing body of evidence has shown that the T7SSb mediates interbacterial competition, through the secretion of polymorphic protein toxins (Chatterjee et al. 2013, Cao et al. 2016, Whitney et al. 2017, Ulhuq et al. 2020, Kobayashi 2021, Klein et al. 2022, Tassinari et al. 2022, Garrett et al. 2023). Polymorphic toxins are commonly used by bacteria in the context of competition, and have a modular arrangement. They comprise a conserved domain responsible for targeting to the appropriate secretion system, and a toxic domain which can carry a range of different toxin functionalities (Zhang et al. 2012). In addition, toxin domains are often highly variable, coevolving with cognate immunity proteins, likely in an attempt to escape immunity mechanisms of competing bacteria (Koskiniemi et al. 2014, Cao et al. 2016, Garrett et al. 2022). Four such polymorphic toxins have now been identified that are associated with the *S. aureus* T7SSb, EsaD, TspA, TslA, and EsxX. Whilst a role for EsxX in interbacterial competition has not been tested, the remaining toxin substrates participate in interbacterial competition and are described below.

## EsaD: a nuclease toxin

**Figure 3.**
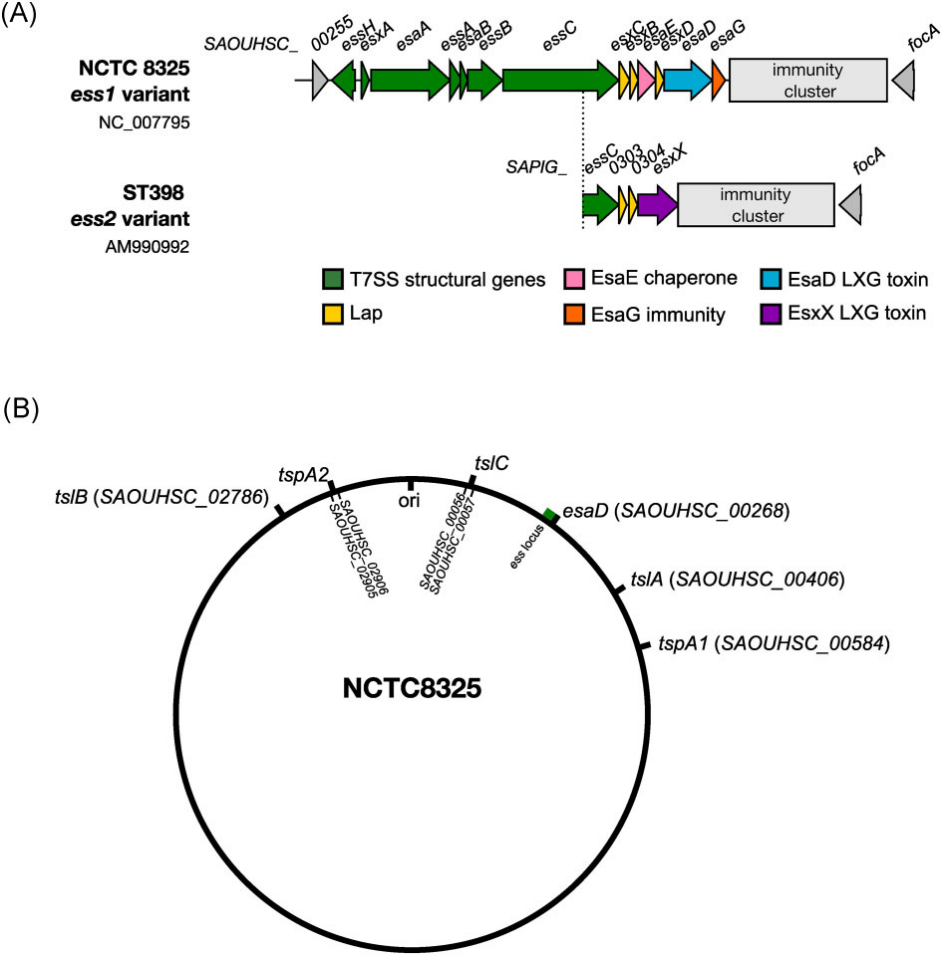
Distribution of known T7SSb toxin-encoding genes on the *S. aureus* chromosome. (A) The *ess*/*T7SSb* locus from NCTC8325 and ST398. A cluster of genes encoding immunity proteins against T7SS toxins is found downstream of the *ess/T7b* locus (grey box). Gene diagrams were visualized using Clinker (Gilchrist and Chooi 2021). (B) Distribution of genes coding for T7SSb LXG substrates on the *S. aureus* NCTC8325 chromosome. The *tslB* gene found in NCTC8325 is annotated as a pseudogene, however, full-length homologues are found in other *S. aureus* strains. Note that *tspA2* and *tslC* are not found in the *S. aureus* NCTC8325 strain. The encoding genes have been included on this figure to indicate the position in which they are found on the *S. aureus* chromosome in encoding strains.

**Figure 4.**
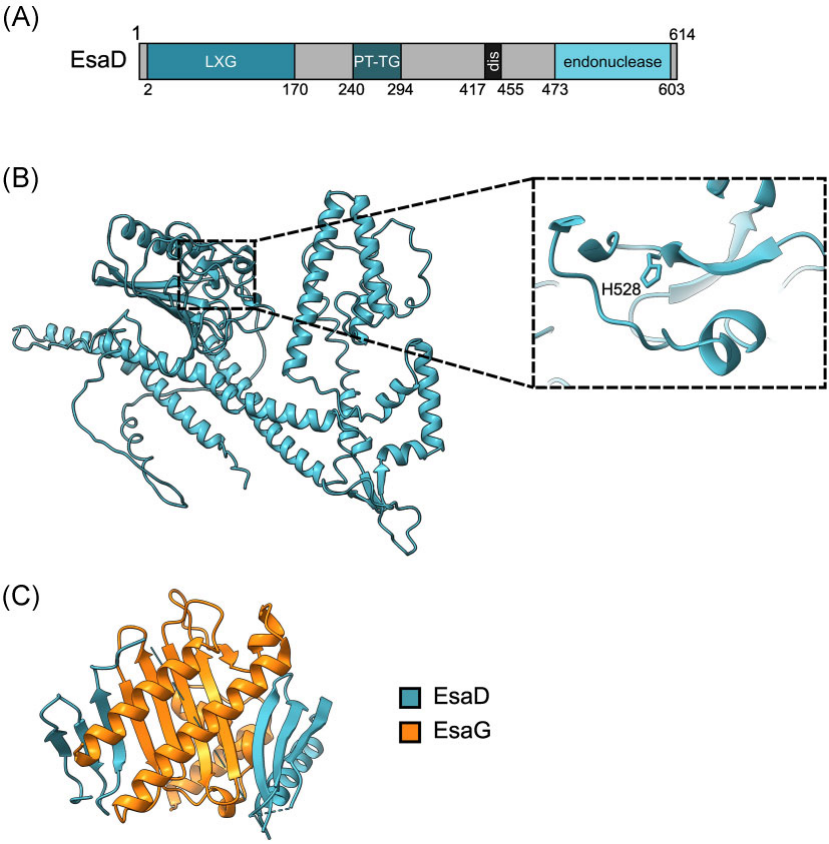
The EsaD substrate of the *S. aureus* T7SS. (A) Domain organization of EsaD predicted by InterProScan (Yang et al. 2023). PT-TG—pretoxin-TG domain; dis—disordered region. (B) An AlphaFold Colab model EsaD (SAOUHSC_00268) from NCTC8325 (Jumper et al. 2021, Varadi et al. 2022). (inset) The active site of the EsaD nuclease domain with the catalytic H528 highlighted. (C) The crystal structure of EsaG (PDB: 8GUO) binding to the EsaD nuclease domain resulting in deformation of the active site. ChimeraX was used to visualize all structural models (Petterson el al. 2020).

EsaD was the first large toxin identified as a substrate of the *S. aureus* T7SSb, encoded downstream of the core components of the secretion system at the *ess/T7SSb* locus (Fig. 3A and B). It should be noted that four variants of the T7SSb have been identified in *S. aureus*, classified based on sequence diversity at the C-terminus of EssC (Warne et al. 2016). EsaD is encoded in *essC1* variant strains, which account for around 50% of *S. aureus* sequenced isolates. The N-terminus of EsaD is composed of an LXG domain followed by a pretoxin-TG (PT-TG) domain (Cao et al. 2016, Yang et al. 2023) (Fig. 4A). PT-TG domains are linker regions carrying a TG motif, of unknown function, that are found in many bacterial toxins. LXG domains, named for the conserved L-x-G residues found in the amino acid sequence (where x is any amino acid; Zhang et al. 2012), are helical and are required for targeting proteins to the T7SSb (Klein et al. 2022). However, LXG domains alone are not competent for secretion by the T7SSb—instead they require interaction with further small helical partner proteins, termed Laps (**L**XG **a**ccessory **p**rotein), to form a presecretion complex (Klein et al. 2022, 2024). EsaD interacts with three small helix-turn-helix Lap proteins, which are necessary for its efficient secretion by the T7SSb. These Lap proteins, EsxB, EsxC, and EsxD, bind to the LXG domain of EsaD, forming the presecretion complex, and are subsequently secreted by the T7SSb along with EsaD (Yang et al. 2023). EsaD also requires a chaperone protein, EsaE for its stability and secretion. EsaE interacts with the central ATPase subunit of the T7SSb, EssC, and may play a role in targeting the EsaD complex to the secretion system (Cao et al. 2016). It remains unclear whether EsaE is secreted with the complex, or if it dissociates during secretion and remains in the cytoplasm (Cao et al. 2016, Yang et al. 2023).

The C-terminus of EsaD encodes a Mg^2+^-dependent nuclease domain with a ββα-metal finger motif, which can degrade double stranded DNA (Cao et al. 2016, Wang et al. 2022) (Fig. 4B). This nuclease domain is exceptionally toxic when expressed in *Escherichia coli*or *S. aureus* (Cao et al. 2016). For self-protection, *S. aureus* produces a cognate immunity protein, EsaG, which is encoded directly downstream of *esaD*. The production of EsaG recovers growth of cells that are producing EsaD by binding directly to the nuclease domain (Cao et al. 2016). EsaG binding disrupts the active site of EsaD by inserting between two structurally important beta-sheets (Wang et al. 2022) (Fig. 4C). This results in distortion of the catalytic site, preventing nuclease activity. *S. aureus* strains also encode strings of nonidentical EsaG homologues, which can diversify by homologous recombination to produce new nonidentical variants (Cao et al. 2016, Garrett et al. 2023). Many of these nonidentical EsaG proteins cannot interact with EsaD encoded in the same strain (Cao et al. 2016). EsaD proteins encoded across *S. aureus* strains have highly polymorphic nuclease domains, suggesting that the role of accessory EsaG proteins is to protect from intoxication by EsaD variants secreted from other bacterial cells.

Whilst the absence of EsaD leads to a reduction in abscess formation in a murine model, EsaD plays no detectable role in virulence using a zebrafish embryo model of infection (Ohr et al. 2017, Ulhuq et al. 2020). EsaD does, however, mediate interbacterial competition. *S. aureus* cells overproducing EsaD were found to outcompete prey cells, which were deleted for the cluster of genes coding for EsaG immunity proteins *in vitro* (Cao et al. 2016). Expression of EsaG in the prey cell recovered their growth, suggesting the deficiency in growth was due to intoxication by EsaD. Similarly, using the zebrafish embryo as a host, *in vivo* competition was observed between wild type *S. aureus* used as the attacker strain and a mutant lacking all EsaG immunity proteins as prey (Ulhuq et al. 2020).

## TspA: a membrane depolarizing toxin

**Figure 5.**
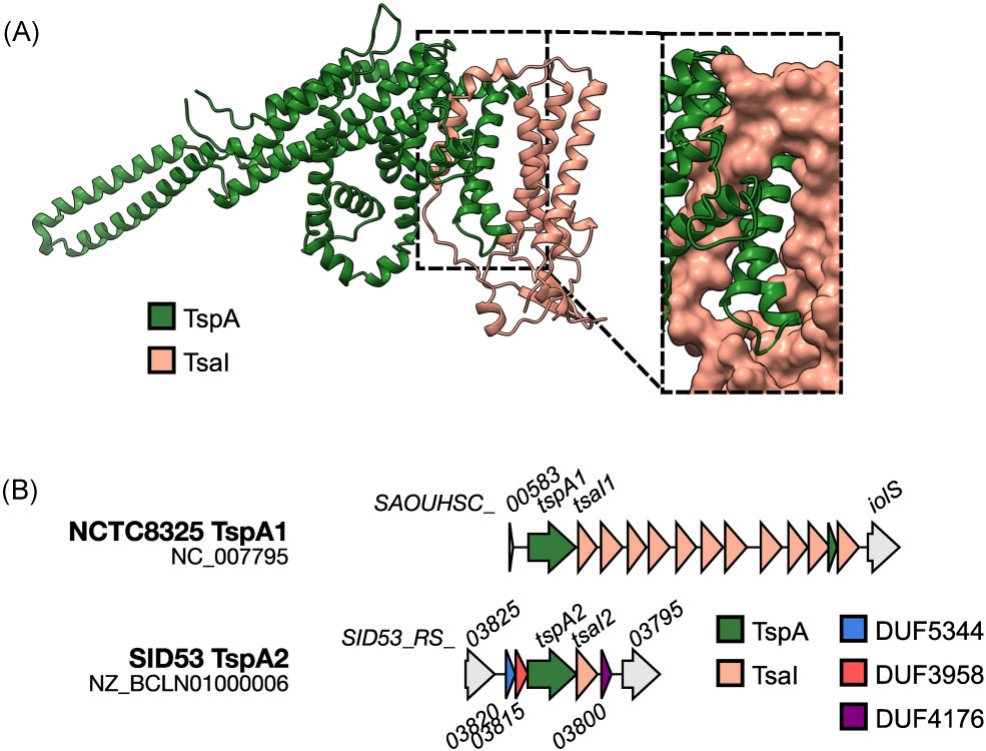
An AlphaFold Colab model of TspA in complex with TsaI. (A) Alphafold Colab was used to model TspA (SAOUHCS_00584) in complex with its immunity protein, TsaI (SAOUHSC_00585) (Jumper et al. 2021, Varadi et al. 2022). (inset) The predicted interaction between TspA and TsaI from *S. aureus* NCTC8325 is highlighted by illustrating the surface of TsaI. ChimeraX was used to visualize all structural models (Petterson el al. 2020). (B) Genetic organization of the *tspA1* and *tspA2* loci in the indicated strains. Gene diagrams were visualized using Clinker (Gilchrist and Chooi 2021).

TspA is another LXG domain-containing protein produced by *S. aureus*. It has a toxic C-terminal domain capable of causing membrane depolarization when it interacts with the extracellular face of the bacterial membrane (Ulhuq et al. 2020). Toxicity is neutralized by coproduction of the membrane-bound immunity protein, TsaI. Although the precise mechanism of rescue is unknown (Ulhuq et al. 2020), an AlphaFold model of TspA with TsaI suggests that TsaI may interact with two predominantly hydrophobic helices from the TspA toxin domain (Fig. 5A), neutralizing TspA activity through complex formation as it inserts into the membrane.

Unlike EsaD, TspA is encoded at a conserved locus, i.e. distant from the *ess/T7SSb* gene cluster and is present in all strains regardless of whether they have the *essC1, essC2, essC3*, or *essC4* variant of EssC, although only secretion by EssC1 has been tested (Fig. 5B; Ulhuq et al. 2020). At this locus, *tspA* is followed by a string of genes coding for TsaI homologues (Cao et al. 2016, Bowman and Palmer 2021a), which vary in number between strains (Garrett et al. 2022). In a handful of *S. aureus* strains, a homologous copy of TspA is encoded at a second locus, distant from both the *ess/T7SSb* and *tspA1* loci (Figs 3B and 5B) (Bowman and Palmer 2021b). At this locus, three small genes coding for domains of unknown function (DUF) are found in an operon with *tspA2* (Bowman and Palmer 2021a). Two of these genes code for small proteins that are predicted to be helical Lap proteins. TelD, an LXG effector from *Streptococcus intermedius*, requires two small Lap proteins for secretion, named LapD1 and LapD2 (Klein et al. 2022). LapD1/2 form a complex with the LXG domain of TelD, facilitating its secretion via the T7SSb (Klein et al. 2022). LapD2 is a DUF3958 protein, the same family as one of the DUF proteins encoded at the *tspA2* locus (Bowman and Palmer 2021a, Klein et al. 2022). Whilst most strains do not encode a copy of *tspA* at this locus, the genes for the three small DUF proteins appear to be conserved, suggesting they could play a role in targeting TspA to the T7SS. This hypothesis remains to be tested (Bowman and Palmer 2021a) (Fig. 5B).

## TslA: an antibacterial ‘reverse’ lipase toxin

All the T7SS antibacterial toxins that have been identified in Bacillota to date show a conserved domain architecture, comprising a C-terminal toxin domain, and an N-terminal LXG or LXG-like domain (Zhang et al. 2011, Cao et al. 2016, Whitney et al. 2017, Ulhuq et al. 2020, Bowman and Palmer 2021a,b, Teh et al. 2023), with the N-terminal domain involved in targeting the effector to the T7SSb, in complex with two or three Lap proteins (Klein et al. 2022, Yang et al. 2023). Recently, a new class of T7SSb substrate has been identified, which has a reversed domain architecture.

**Figure 6.**
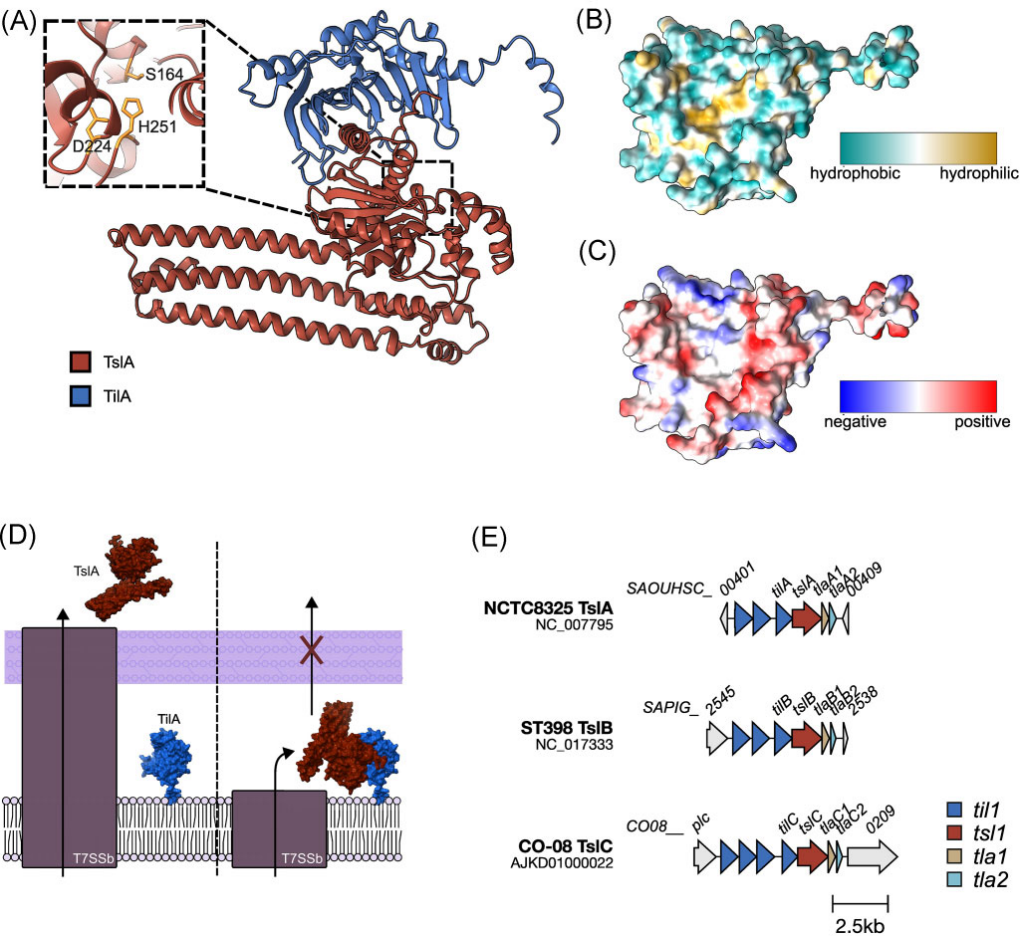
TilA immunity protein binding to TslA. (A) An AlphaFold Colab model of TslA in complex with its immunity protein, TilA (Jumper et al. 2021, Varadi et al. 2022). (Inset) The catalytic triad of TslA is composed of S164, D224, and H251 in TslA (SAOUHSC_00406) from *S. aureus* NCTC8325. ChimeraX was used to visualize all structural models (Petterson el al. 2020). (B) and (C) An AlphaFold Colab model of TilA with (B) lipophilicity and (C) electrostatic potential mapped onto the surface. (D) A model of TslA export out of the cell if the T7SS spans the entire cell wall (left) or if the T7SS spans only the cytoplasmic membrane (right). (E) Genetic organization of the *tslA, tslB*, and *tslC* loci in the indicated strains. Gene diagrams were visualized using Clinker (Gilchrist and Chooi 2021).

TslA is the first ‘reverse’ substrate of the T7SSb to be characterized. It has a helical LXG-like domain similar to other T7SSb substrates, but in TslA this is found at the C-terminus rather than the N-terminus (Garrett et al. 2023) (Fig. 6A). Nevertheless, it has been shown that this LXG-like domain is required for secretion of TslA by the T7SSb. Secretion is facilitated by the binding of two small Lap proteins to this domain, as has been observed for other T7SSb substrates (Klein et al. 2022, Yang et al. 2023, 2024). This suggests that the T7SSb can recognize a secretion signal present at either end of a substrate protein, which is a highly unusual feature for a protein secretion system.

TslA has phospholipase A activity and can cleave a wide range of bacterial membrane phospholipids, mediated by its N-terminal lipase domain (Garrett et al. 2023) (Fig. 6A). This results in accumulation of lyso-acyl phospholipids, which have detergent-like properties and lead to disruption of the cell membrane. This significantly perturbs the growth of susceptible *S. aureus* cells. Toxicity can be abrogated by the production of a lipidated immunity protein, TilA, embedded in the outer leaflet of the cytoplasmic membrane (Garrett et al. 2023). TilA binds with 14.2 nM affinity to the N-terminal lipase domain of TslA to inhibit this activity. It is unclear how the two proteins interact with one another, however, an AlphaFold Colab model of TilA indicates that there are hydrophobic, hydrophilic, and charged residues present on the concave face, which may be involved in this interaction (Fig. 6B and C).

Whilst the mechanism of secretion of substrates by the T7SSb is still not understood, it has been proposed that substrates are translocated across the entire cell envelope in a single step. The crystal structure of the large extracellular loop of the EsaA T7SSb component of *Streptococcus* reveals that it is of sufficient length to fully span the peptidoglycan cell wall (Klein et al. 2018, 2021). Given that TilA binding to TslA is very tight, it suggests that during secretion, the toxin must bypass the periplasm environment where the TilA immunity protein resides as complex formation would result in the toxin being retained by the producing cell (Fig. 6D).

Homologues of TslA can be encoded at up to two additional loci on the *S. aureus* genome, depending on the particular strain (Garrett et al. 2023) (Figs 3B and 6E). Whilst these homologues have not been studied to date, their conservation suggests that these ‘reverse’ toxins make up a significant part of the *S. aureus* T7SSb toxin repertoire. Unlike bacteriocins, which appear to be sporadically encoded by *S. aureus*, the T7SSb is encoded by all strains, suggesting this represents a widespread competitive mechanism. Moreover, both TslA and TspA, or homologues of these, are encoded almost ubiquitously by *S. aureus*. Although EsaD is only carried by strains, which harbour the *essC1* variant of the T7SSb (Warne et al. 2016), other *essC* types encode putative LXG or LXG-like toxins at their T7SSb loci, which are predicted to have similar antibacterial activities (Bowman and Palmer 2021b). This includes EsxX, which is encoded downstream of *essC* in *essC2* variant strains (Fig. 1A). The toxin domain of EsxX has been proposed to share structural similarity with colicin IA, indicating that it may be pore-forming (Dai et al. 2017). Further work of EsxX is required to understand the role it may play in interbacterial competition.

Future studies should begin to shed light on how these toxins are delivered to the target cell, and in the case of intracellularly acting toxins such as EsaD, to provide an understanding of how they access the cytoplasm of target bacteria. A role in virulence has also been cited for EsaD, EsxX, and TspA (Dai et al. 2017, Ohr et al. 2017, Ulhuq et al. 2020), at least for some strains and virulence models. Whilst no statistically significant role in murine skin abscess formation was observed for TslA, there was a trend towards reduced virulence when the encoding gene was deleted, suggesting that there may be a cumulative effect of these secreted proteins on the host (Garrett et al. 2023). Further work into understanding toxin delivery to targets will provide important context to these phenotypes, as there is currently a gap in knowledge between the virulence phenotypes associated with the T7SS of *S. aureus* and the molecular mechanisms by which they work.

## Concluding remarks

In recent years bioinformatic studies have identified additional putative polymorphic toxin systems across a broad spectrum of bacterial species, including in *S. aureus* (Zhang et al. 2012, Li et al. 2022), suggesting there are further antibacterial compounds that may be used by *S. aureus* in a competition setting. Nevertheless, there are two major mechanisms currently described that are employed by *S. aureus*. The T7SS is responsible for the secretion of several polymorphic toxins, which are known to kill other *S. aureus* species in the absence of a cognate immunity protein. Bacteriocins produced by *S. aureus* have been shown to inhibit the growth of a diverse range of bacterial species, however, appear to be less conserved across *S. aureus* strains in comparison to the highly conserved T7SS. These mechanisms of interbacterial competition likely work in tandem to control competitors in the polymicrobial environments that they colonize. As *S. aureus* strains of different clonal complexes are commonly found to colonize different niches (Howden et al. 2023), understanding the distribution of bacteriocins and the polymorphic T7SS toxins in the context of *S. aureus* lineage may illuminate the predominant role of some of these compounds in interbacterial competition.

Some *S. aureus*-produced bacteriocins show great promise in the production of alternatives to antibiotics, however, much work is still need to determine the molecular mechanism of killing employed by many of these bacteriocins and to further assess the possibility of resistance development in bacterial populations. The resistance mechanisms utilized against bacteriocins are often associated with the presence of the bacteriocin gene cluster, with transporters usually providing resistance to toxicity (Ennahar et al. 2000, Gajic et al. 2003, Ishibashi et al. 2014). However, it has been found that escape mutations in target cells, including in their two-component systems and ABC transporters, can provide resistance to bacteriocin killing (Tymoszewska et al. 2021, 2023). For toxins secreted by the T7SS, strings of immunity proteins are commonly found in both producing and closely related, T7SS-deficent bacterial species, implying a continual coevolution between toxin and immunity genes (Bowman and Palmer 2021, Garrett et al. 2022). As such, a firm understanding of acquired resistance to these compounds is essential for the development of such proteins for therapeutic uses.

The *S. aureus* T7SSb has been shown to play roles in both virulence and interbacterial competition. As further research uncovers the molecular mechanisms of protein secretion and the role of secreted substrates it is anticipated that we will have a clearer understanding of how this system contributes to virulence and colonization. It will be interesting to decipher how antibacterial toxins traverse the envelope of susceptible cells to access their molecular targets. The continued work on *S. aureus*-produced antibacterial molecules will hopefully provide a clearer understanding of how this major human pathogen has evolved to colonize the host with such success.

## Supplementary Material

xtae006_Supplemental_Files
