# Supplementary material for: The role of proteinaceous toxins secreted by Staphylococcus aureus in interbacterial competition
Source: FEMS Microbes. 2024 Feb 28;5:xtae006. doi: 10.1093/femsmc/xtae006 (PMC10941976; doi:10.1093/femsmc/xtae006)
Supplement: xtae006_Supplemental_Files [file xtae006_supplemental_files.zip › FEMSMC-2023-041.r1_supplementary_data_-_reviews_and_author_response.docx]

**Reviews of manuscript FEMSMC-2023-041**

**Reviewer 1**

**Recommendation: Minor Revision**

This manuscript is a comprehensive review of antibacterial compounds produced by S. aureus, an important pathogen as well as a commensal. The ability of S. aureus to survive in host niches is a critical prerequisite for the organism to colonize and cause infections. The antibacterial compounds mediate inter- and intra-species competition and are therefore important for S. aureus survival and modulation of its niche environment. This review covers different types of S. aureus bacteriocins, PSMs, and T7SS-secreted toxins that have not been extensively reviewed previously and therefore provides valuable information for the research community.

The manuscript is clearly written and easy to read. I have only a few concerns.

Class III, V and VI bacteriocins are mentioned in the summary section of S. aureus secreted bacteriocins (lines 73-75 for III, and 78-85 for V and VI), but do not have separate sections describing individual bacteriocins, as for the other classes. In the abstract, the authors mentioned that only class I, II and IV are produced. This is a little confusing. Can the authors make it clear in the “S. aureus secreted bacteriocins” section whether these classes are produced by S. aureus or not.

The authors described the in vivo effect of EsaD. Are there any studies that examined the role of bacteriocins, PSMs or other T7SS-secreted toxins in vivo, e.g., contribution to colonization or virulence? Can the authors describe or comment (if lacking) on this in the manuscript?

Fig 2 summarizes the bacteriocins and their target range. It will be nice to have a figure(s) listing the target range for PSMs and T7SS-secreted toxins.

**Reviewer 2: Anne Jamet, INSERM**

**Recommendation: Major Revision**

In this review, Garrett et al describe the protein toxins secreted by Staphylococcus aureus as part of interbacterial competition. While interesting, the article would benefit from the following changes to improve scientific accuracy and clarity:

1-I suggest adding more general brief paragraphs to avoid catalogs and scattered redundant information.

In the "Introduction" section:

- A summary of the means of identification/purification (supernatant activity, known genes, bioinformatics study...) with a historical perspective

- general concepts of self-protection of producing strains (immunity) and post-segregational killing

- known export systems

- modularity and polymorphic organization of toxins (10.1128/mbio.00285-15)

In the "Perspective" section:

- potential industrial and medical use

- risk of potential resistance, cross-immunity

- brief discussion of physiological role (competition, post-segregational killing...)

2-The choice to use the terms bacteriocin, inhibitory substance and BLIS is unclear. Could you add definitions and improve consistency throughout the manuscript?

3-Minor changes:

Line 30: S. aureus could be better described as "opportunistic pathogen".

Line 34: S. aureus infections most often require skin damage or a pre-existing medical condition.

Line 107: Can you be more specific about the cleavage of PSMs?

Line 231: I would delete "like this".

Lines 231-233: Move this sentence into a general discussion of therapeutic potential and resistance.

Lines 310-319: move this part to the paragraphs devoted to introduction (means of identification, physiological roles) and perspective (industrial/medical use).

Lines 371-373: the 4 variants of the T7SS should be introduced first in the general introductory section of the T7SS.

Lines 488-489: This information should appear in an introductory paragraph.

Figure 2: could be extended with PSM and T7SS effectors

**Reviewer 3**

**Recommendation: Minor Revision**

The authors provide an excellent and comprehensive review of Staphylococcus aureus bacteriocins/T7SS toxins that mediate interbacterial competition. It is well-written and will serve as a helpful resource on mechanisms of staphylococcal competition during colonization of the host. Below I highlight points that could benefit from further elaboration as well as minor comments.

• While there is obviously a great deal of heterogeneity, It would be helpful to provide more introductory information in the main text/figure on any conserved aspects of bacteriocin synthesis, export/transport, mechanisms of action of classes I-IV (static vs cidal), modification, regulation, and/or neutralization by immunity factors, amino acid composition, etc.

• Figure 2 may be slightly misleading in that all the target species listed were likely not tested in each study. Can the authors distinguish in this figure which species were truly resistant/sensitive to a given bacteriocin, vs species against which a given bacteriocin was not tested?

• The bacteriocin section of this review highlights S. aureus competition with non-kin species/genera, but the section on T7SS toxin-mediated competition exclusively refers to intra-species S. aureus killing. Can the authors address whether there is any indication that S. aureus T7SS toxins may mediate competition with other bacteria?

• It would be helpful to provide more information on the relationship between S. aureus clades/lineages and the number/classes of bacteriocin(s) encoded. Do some S. aureus strains encode multiple bacteriocins, and if so, can a given strain produce multiple bacteriocins of different class types? Is there a difference in the number/variety/class of bacteriocin produced by colonizing isolates vs invasive isolates?

• Line 480-483. The TslA/TilA mechanism described here is a little unclear. Can the authors clarify or provide a cartoon figure to better explain this mechanism?

Minor:

• Line 43-58: It should be acknowledged that S. aureus also commonly colonizes the skin and vaginal tract, additional polymicrobial niches in which interbacterial competition is relevant.

• Line 55: “aureus” should be italicized.

• Line 59-60: Please provide a citation of where this information has previously been reviewed.

• Line 18-19, 86-92: can the authors clarify the difference between “bacteriocin” vs “bacteriocin like substance”. Is the only difference that “BLIS’ have unknown genetic origin?

• Line 95-110: It would be helpful to describe the genes encoded in the bacteriocin loci in the main text, instead of just in the Figure 1 legend. Specifically, in the Bsa locus, what are bsaB-G predicted to encode if not transporters, regulators, immunity factors, etc? Bsa/Au-26 is stated in the text to have activity against Strep (lines 95-98), but this is not reflected in the shading in Figure 2.

• Line 115: Please define the “ET3-1” of BsaA2ET3-1.

• Lines 114, 138-144. The designation of class I vs II bacteriocins as post-translationally modified vs unmodified, respectively (lines 69-73), is slightly confusing as examples are given later in the text (Class I BacCH91 and Class II Cssɑ/β) that do not appear to follow this rule.

• Lines 202-205: It is not clear how presence/absence of bacteriocin synthesis/regulator genes on a plasmid is related to bacteriocin post-translational modifications/structure. Can the authors clarify these sentences?

• Line 228-230: two component systems are mentioned to promote aureocin resistance. Were specific genes within those TCS regulons identified that may more specifically promote resistance and can the authors elaborate on the potential for development of bacteriocin resistance in general?

• Can the authors further comment on the evolution of this wide variety/heterogeneity of bacteriocins in contrast to the highly conserved T7SS toxins?

• Line 335-336. Please define “Pmt” and elaborate briefly on its function.

• Line 429-443. Has TspA been shown to be secreted by all 4 EssC variants? Has TspA1 been shown to interact with the Laps encoded for near tspA2?

• Line 471-472 and 480-481 indicate that binding of TslA to its immunity factor is “tight”. Please indicate the affinity of this interaction.

• Line 494-495: can the authors elaborate slightly on EsxX? I understand it has not yet been studied in terms of competition, but does its contain known toxic domains? Are EsxX+ strains typically isolated from different niches compared to EssD+ strains?

**Editor Decision: Major Revision**

**Author's Response to Decision Letter for (FEMSMC-2023-041)**

We thank the editor for handling our manuscript and the reviewers for their careful reviews and helpful comments. Below we respond to each point raised, in reviewer order, with our responses indicated by an arrow. We have uploaded an unmarked copy of the revised manuscript and a marked copy using track changes. All line numbers in our response relates to the *unmarked* copy.

Yours sincerely,

Stephen Garrett pp all authors

Reviewer: 1

Comments to the Author

This manuscript is a comprehensive review of antibacterial compounds produced by S. aureus, an important pathogen as well as a commensal. The ability of S. aureus to survive in host niches is a critical prerequisite for the organism to colonize and cause infections. The antibacterial compounds mediate inter- and intra-species competition and are therefore important for S. aureus survival and modulation of its niche environment. This review covers different types of S. aureus bacteriocins, PSMs, and T7SS-secreted toxins that have not been extensively reviewed previously and therefore provides valuable information for the research community.

We thank the reviewer for their positive comments.

The manuscript is clearly written and easy to read. I have only a few concerns.

Class III, V and VI bacteriocins are mentioned in the summary section of S. aureus secreted bacteriocins (lines 73-75 for III, and 78-85 for V and VI), but do not have separate sections describing individual bacteriocins, as for the other classes. In the abstract, the authors mentioned that only class I, II and IV are produced. This is a little confusing. Can the authors make it clear in the “S. aureus secreted bacteriocins” section whether these classes are produced by S. aureus or not.

We apologise for the ambiguity of this section. Whist some of these are produced by other staphylococci, we have not found any evidence in the literature for Class III, V or VI bacteriocin production by S. aureus.

We have now clarified this in text (lines 111-112).

The authors described the in vivo effect of EsaD. Are there any studies that examined the role of bacteriocins, PSMs or other T7SS-secreted toxins in vivo, e.g., contribution to colonization or virulence? Can the authors describe or comment (if lacking) on this in the manuscript?

Some virulence studies have been carried out for other T7SS-substrates and the major role of PSMs is believed to be in virulence.

As this isn’t the main focus of this review, we have added a short comment for each directing the reader to studies regarding virulence of the discussed proteins. (lines 536-547; 347-348).

Fig 2 summarizes the bacteriocins and their target range. It will be nice to have a figure(s) listing the target range for PSMs and T7SS-secreted toxins.

We think this is a great suggestion and may form a nice area of future study, however, there is not currently enough evidence in the literature to support such a figure. The antibacterial effects of PSMs remain poorly studied and the target range of T7SS-substrates secreted by S. aureus remains unknown, with toxicity only currently tested against immunity-deficient S. aureus strains.

Reviewer: 2

Comments to the Author

In this review, Garrett et al describe the protein toxins secreted by Staphylococcus aureus as part of interbacterial competition. While interesting, the article would benefit from the following changes to improve scientific accuracy and clarity:

We thank the reviewer for the thorough review and the helpful suggestions to improve the manuscript.

1-I suggest adding more general brief paragraphs to avoid catalogs and scattered redundant information.

In the "Introduction" section:

- A summary of the means of identification/purification (supernatant activity, known genes, bioinformatics study...) with a historical perspective

- general concepts of self-protection of producing strains (immunity) and post-segregational killing

- known export systems

- modularity and polymorphic organization of toxins (10.1128/mbio.00285-15)

In the "Perspective" section:

- potential industrial and medical use

- risk of potential resistance, cross-immunity

- brief discussion of physiological role (competition, post-segregational killing...)

While we understand the formatting suggests made by the reviewer, we feel like the structure of this review article best portrays the information we wish to summarise.

We appreciate some of the suggestions made will improve accessibility for the reader, which we have addressed: We have now added a broader discussion on resistance mechanisms to both bacteriocins and T7SS toxins, including immunity mechanisms and export systems (lines 569-581). We have also introduced the concept of polymorphic toxins in greater details (lines 390-396). We have not expanded on therapeutic potential of these bacteriocins as, whilst we acknowledge this is an important area of research, it does not inform the focus of this review which is interbacterial competition in S. aureus.

2-The choice to use the terms bacteriocin, inhibitory substance and BLIS is unclear. Could you add definitions and improve consistency throughout the manuscript?

We have now clarified the distinction between bacteriocins and BLIS (line 108-109). In the paragraph discussing BLIS, inhibitory substances is used in the opening of that paragraph to encompass a broader range of inhibitory compounds, including antibiotics etc.

3-Minor changes:

Line 30: S. aureus could be better described as "opportunistic pathogen".

This has now been updated (lines 37; 52).

Line 34: S. aureus infections most often require skin damage or a pre-existing medical condition.

We have now included this (line 42).

Line 107: Can you be more specific about the cleavage of PSMs?

This is discussed in the PSM paragraph later in the manuscript, so has been removed to avoid confusion. (Line 130-131).

Line 231: I would delete "like this".

We have now removed this.

Lines 231-233: Move this sentence into a general discussion of therapeutic potential and resistance.

Whilst we understand that the therapeutic potential of bacteriocins is important, the focus of this review is interbacterial competition mediated by proteins secreted by S. aureus. This is why we have mentioned therapeutic potential in passing but have not focused on it in this review.

Lines 310-319: move this part to the paragraphs devoted to introduction (means of identification, physiological roles) and perspective (industrial/medical use).

While we understand the formatting suggestions made by the reviewer, we feel like the structure of this review article best portrays the information we wish to summarise.

Lines 371-373: the 4 variants of the T7SS should be introduced first in the general introductory section of the T7SS.

We feel that moving this to the introduction will make the section disjointed and harder for the reader to follow so have chosen to keep the T7SS variants discussion in this paragraph.

Lines 488-489: This information should appear in an introductory paragraph.

This is already present in the T7SS introductory paragraph (lines 367-368); however, we have now expanded on the comparisons in the concluding paragraph (lines 557-565).

Figure 2: could be extended with PSM and T7SS effectors

As mentioned in response to Reviewer 1, we think this will form an interesting area of future study, however, due to the lack of target strains tested for both PSM and T7SS toxin targeting, we don’t believe that this will be informative in Figure 2 as a very limited range of strains have been tested.

Reviewer: 3

Comments to the Author

The authors provide an excellent and comprehensive review of Staphylococcus aureus bacteriocins/T7SS toxins that mediate interbacterial competition. It is well-written and will serve as a helpful resource on mechanisms of staphylococcal competition during colonization of the host. Below I highlight points that could benefit from further elaboration as well as minor comments.

We thank the reviewer for their thorough review and constructive comments on the manuscript.

• While there is obviously a great deal of heterogeneity, It would be helpful to provide more introductory information in the main text/figure on any conserved aspects of bacteriocin synthesis, export/transport, mechanisms of action of classes I-IV (static vs cidal), modification, regulation, and/or neutralization by immunity factors, amino acid composition, etc.

We have now expanded the introduction to include more detail on bacteriocin synthesis and transport (lines 77-86).

• Figure 2 may be slightly misleading in that all the target species listed were likely not tested in each study. Can the authors distinguish in this figure which species were truly resistant/sensitive to a given bacteriocin, vs species against which a given bacteriocin was not tested?

We have now update figure 2 to indicate which bacterial species were tested for each bacteriocin.

• The bacteriocin section of this review highlights S. aureus competition with non-kin species/genera, but the section on T7SS toxin-mediated competition exclusively refers to intra-species S. aureus killing. Can the authors address whether there is any indication that S. aureus T7SS toxins may mediate competition with other bacteria?

Competition mediated by the T7SS of S. aureus has, to date, only been shown intra-species and has not been described in the literature as having activity against other bacterial species. The T7SS of Streptococcus intermedius was found to have activity against a range of Gram positive organisms and so, whilst it is possible that the S. aureus T7SS may display a similar phenotype, this remains to be tested.

• It would be helpful to provide more information on the relationship between S. aureus clades/lineages and the number/classes of bacteriocin(s) encoded. Do some S. aureus strains encode multiple bacteriocins, and if so, can a given strain produce multiple bacteriocins of different class types? Is there a difference in the number/variety/class of bacteriocin produced by colonizing isolates vs invasive isolates?

Whilst there is some evidence in the literature of S. aureus strains carrying multiple bacteriocins, an extensive study into this has not been performed and so we cannot comment on this in this review, however we acknowledge this would be an interesting line of future investigation to better understand interbacterial competition in S. aureus.

• Line 480-483. The TslA/TilA mechanism described here is a little unclear. Can the authors clarify or provide a cartoon figure to better explain this mechanism?

We have now included an additional panel on Fig 6 to better describe this mechanism.

Minor:

• Line 43-58: It should be acknowledged that S. aureus also commonly colonizes the skin and vaginal tract, additional polymicrobial niches in which interbacterial competition is relevant.

We have now incorporated this into the introduction (lines 54-55).

• Line 55: “aureus” should be italicized.

This has been corrected.

• Line 59-60: Please provide a citation of where this information has previously been reviewed.

This has been added (line 69-70).

• Line 18-19, 86-92: can the authors clarify the difference between “bacteriocin” vs “bacteriocin like substance”. Is the only difference that “BLIS’ have unknown genetic origin?

We have now clarified this in text (line 110-113).

• Line 95-110: It would be helpful to describe the genes encoded in the bacteriocin loci in the main text, instead of just in the Figure 1 legend. Specifically, in the Bsa locus, what are bsaB-G predicted to encode if not transporters, regulators, immunity factors, etc? Bsa/Au-26 is stated in the text to have activity against Strep (lines 95-98), but this is not reflected in the shading in Figure 2.

We have now expanded on this in text (lines 124-126). We have also corrected the activity profile of Bsa/Au-26 in Fig 2.

• Line 115: Please define the “ET3-1” of BsaA2ET3-1.

Clarified in text (line 139).

• Lines 114, 138-144. The designation of class I vs II bacteriocins as post-translationally modified vs unmodified, respectively (lines 69-73), is slightly confusing as examples are given later in the text (Class I BacCH91 and Class II Cssɑ/β) that do not appear to follow this rule.

We apologise for the confusion as we had incorrectly classified the C55 bacteriocin pair as Class II rather than Class I. This has now been corrected in text (line 154)

We have also corrected BacCH91, which was found to be post-translationally modified (line 38).

• Lines 202-205: It is not clear how presence/absence of bacteriocin synthesis/regulator genes on a plasmid is related to bacteriocin post-translational modifications/structure. Can the authors clarify these sentences?

We apologise, the word regulation was used in error and has now been corrected (line 226).

• Line 228-230: two component systems are mentioned to promote aureocin resistance. Were specific genes within those TCS regulons identified that may more specifically promote resistance and can the authors elaborate on the potential for development of bacteriocin resistance in general?

These mutations were found in the genes encoding KinG and LlrG which make up the TCS and in the ABC transporter YsaCB. We have now expanded on the discussion on the development of resistance (lines 573-575).

• Can the authors further comment on the evolution of this wide variety/heterogeneity of bacteriocins in contrast to the highly conserved T7SS toxins?

We have now expanded on this discussion (line 557-565).

• Line 335-336. Please define “Pmt” and elaborate briefly on its function.

This has been expanded clarified in text (line 361-362).

• Line 429-443. Has TspA been shown to be secreted by all 4 EssC variants? Has TspA1 been shown to interact with the Laps encoded for near tspA2?

TspA1 secretion has not been tested in other EssC variants and to date has not been shown to interact with the Laps. We have now clarified in text that this is hypothetical (line 481).

• Line 471-472 and 480-481 indicate that binding of TslA to its immunity factor is “tight”. Please indicate the affinity of this interaction.

This has been added in text (line 508-509).

• Line 494-495: can the authors elaborate slightly on EsxX? I understand it has not yet been studied in terms of competition, but does its contain known toxic domains? Are EsxX+ strains typically isolated from different niches compared to EssD+ strains?

We have now expanded on this in text (line 533-535).

Whilst it would be interesting to map the niche from which each T7SS variant are isolated, unfortunately there is extensive sequencing bias for virulent S. aureus strains, and so a conclusive study into this would be difficult.
